# Demand for long acting and permanent methods of contraceptives and factors for non-use among married women of Goba Town, Bale Zone, South East Ethiopia

**DOI:** 10.1186/1742-4755-9-26

**Published:** 2012-10-29

**Authors:** Abulie Takele, Getu Degu, Mezgebu Yitayal

**Affiliations:** 1 College of Medicine & Health Sciences, Madawalabu University, Robe, Bale, Ethiopia; 2 School of Public Health, Gondar College of Medical Sciences, University of Gondar, Gondar, Ethiopia; 3 School of Public Health, Gondar of University, Gondar, Ethiopia

**Keywords:** Long Acting and Permanent Methods, Demand, Unmet need, Utilization, Implants, Contraception

## Abstract

**Background:**

Contraceptive use including short acting, long acting and permanent methods positively influence the socio-economic development of a nation by allowing families to space and limit their family size to their economic capacity. Demand for LAPMs of contraception as detrmined by utilization and unmet need for LAPMs of contraception can provide realiable information for providers.

**Objective:**

To determine the utilization of long acting and permanent contraception and its associated factors among married women of Goba town, South East Ethiopia.

**Methods:**

A cross sectional community based study was conducted among 734 systematically selected married women of reproductive age in Goba town in September/ 2009. A structured and pretested, interview questionaire was used to collect data on socio-demographic, behavioral factors and data related to demand for LAPMs of contraception. Data were analyzed using EPI INFO and SPSS version 16.

**Result:**

The demand for Long Acting and Permanent Methods (LAPMs) of contraception was 18.1%. Utilization of LAPMs of contraception in the town was 64 (8.7%) and the unmet need for LAPMs was 69 (9.4%). Information on LAPMs in the town was 636 (86.6%). Media (radio and television) was the major sources of information 641 (87.3%). The use of LAPMs was significatly associated with ever use AOR[17.43, 95% CI:9.19, 33.03], number of times discussions made on methods AOR[4.6, 95% CI: 1.72,12.17] and main decider of using methods AOR[ 2.2, 95% CI:1.03, 4.65]. It was not associated with socio-demographic variables.

**Conclusion and recommendation:**

The utilization of LAPMs in the town was less although higher than the Ethiopian demographic and health survey 2005 result. Moreover, there was a considerable unmet need. Increase the method mix of LAPMs by incorporating varaies of implnats in order to increase utilization. Proper counseling of client and partners discussion were some of the recommendation forwarded.

## Background

The international conference on population and development held in Cairo in 1994, drew the attention of international community and national governments to the need to improve reproductive health services especially in low economic status countries like Ethiopia [1].

Reproductive-age women have varying contraceptive needs. For many women, long-term reversible contraception, defined as Intra Uterine Devices (IUDs) and implants represents an excellent choice [2-4]. Researchers have showen that many women do not use oral contraceptives effectively. One million pregnancies result from the faulty use of oral contraceptives each year; in addition, unintended pregnancies remain common. Clearly, the method of contraception must tailor to meet the needs of such individuals [3,4]. Long-acting and Permanent Methods of Contraception (LAPMCs) remain relatively small and sometimes missing component of national Family Planning(FP) programs in Sub Saharan Africa (SSA). These methods can enhance FP programs in meaningful ways if the challenges to their availability, access, and acceptability can be overcome [3].

Family Planning service that provides accurate and complete information about contraceptive methods meets the need of their clients. Still, early marriage and producing too many children, which are close to each other is a common practice of developing countries including Ethiopia. The total fertility rate (TFR) is 4.8 nationally and 5.6 in Oromia region. Long Acting and Permanent Methods of contraception use have lowest rates. It account IUD 0.3%; implant 3.4% and female sterilization 0.5%. The contraceptive prevalence rate at national level was 28.6%. The unmet need for family planning was 25% [2]. Hormonal implants are frequently under-utilized FP methods [3,4]. Knowledge of LAPMCs in Oromia region was IUD 12.2%, Implants 20% and Tubal ligation 17.2%. Ever use and current use of Long Acting and Permanent Methods (LAPMs) of contraception in oromia region was less than one percent [5].

Determining the utilization and unmet need for LAPMs of contraceptives indicates demand for contraception by indicating potential users of LAPMs of contraceptives. In the study area, separate study was not conducted to determine demand for LAPMs contraceptives. In order to address this problem it is important to study the factors associated and make the services available at the lowest possible level. Moreover, this study focuses on assessment of the demand for LAPMs of contraceptives and factors for non-utilization among married women of reproductive age in Goba town.

## Methodology

### Study setting and sample

The study was conducted in Bale Zone Goba Town located at 450 km from Addis Ababa in the South East Ethiopia. With a total area of 1619.38 sq.km, population of 32,916, population density of 20.34 people per sq. km and household size of 4.2 people per household and 7,837 household in two sub-cites. A cross sectional community based study conducted in Goba town in September 2009 to assess the demand of LAPMs contraception and factors for non-use among married women of Goba town. The source population was all married women found in Goba town aged 15–49 years. The study population was married women aged 15–49 years found in the three randomly selected Kebeles of Goba town.

A sample of 734 married women of reproductive age was calculated using a proportion of 50%, a type 1 error 5%, a power of 80% and a design effect of two. The town was divided in to six kebele and three of six kebeles were selected by lottery method. Considering at least there is one married woman present per household, and using the number of households as sampling frame. Systematic random sampling method used to take proportional numbers of women from three kebeles. Interview was made by face to face interviewing of married women from the selected kebeles.

### Measurements

A structured and pre- tested interview based questionnaire was used to collect the quantitative data. The questionnaire was comprised of questions for assessing socio-demographic, social, behavioral and service factors were prepared. Some of the questions were taken from EDHS and modified. Trained diploma holders female data collectors collected the data by interviewing wives in the three kebeles. Questionnaire was first prepared in English and translated to Amharic to make it understandable by the study subjects. Data collectors explained the purpose of the study and obtaining verbal consent from the respondent. The data collectors have checked for completeness and consistency before leaving the interviewee.

### Data analysis

Data was analyzed using EPI-INF 6 and exported to SPSS version 16. Recoding and transforming of some data (age of respondent, number of pregnancy, number of children alive, religion, educational status, occupation, monthly income, husband opinion on partners using LAPMs of contraceptives, number of times discussion on LAPMs and main decider on using LAPMs of contraceptives) was done to make them categorical. Descriptive statistics computed to determine the prevalence of LAPMs of contraception use. Analysis such as proportion, percentage, frequency distribution, statistical methods logistic regression, odds ratio with 95% confidence interval, and P-Value used in describing the data. The results were displayed using tables. Association of socio-demographic and other variables were determined using cross tabulating, and odds ratio (OR) with 95% confidence intervals. Stepwise logistic regression was used to control for confounding.

### Ethical clearance

Letter of ethical clearance was obtained from Gondar University School of Public Health Ethical Research Committee. Formal letter of cooperation was written from Gondar University School of Public Heath to Goba town administrative council. Verbal consent was obtained from the respondents and response of participants was anonymous and confidential.

### For uniformity of understanding of the approach of key words is given below

• *Demand for LAPMs:*- The sum of LAPM being used (Met need) and method that is desired but not used due to any reason (unmet need).

• *Long acting contraceptive*: - Intra-Uterine Device (IUD) and Norplant.

• *Permanent methods of contraceptive*- Tubal ligation

• *Unmet need for LAPMs of Contraceptive*:- A condition of wanting to postpone or avoid pregnancy but not using any of the LAPMs.

• *Unmet need for LAPMs of contraceptive during pregnancy: *- A phrase to describe a woman who is pregnant at the time of the study but whose pregnancy was not intended.

• *Unmet need for limiting:*- When women who do not want any more children, but not using any of LAPM or others methods.

• *Unmet need for spacing:*- When a woman who does not want to have pregnancy soon after delivery or want to space for two years but not using any of LAPM or other methods.

## Results

### Socio-demographic characteristics of the respondents

The response rate for the study was 100%. The mean age of the respondents was 30 ± 7.1 S.D and the median age was 29 years. Majority of the respondents 596 (81.2%) were in the age group 20–39 years. Three hundred forty three 46.7% of the respondents had high school education, 32 (4.4%) have informal education and 44 (6%) have no education. The ethnicity of the respondents was Oromo 350 (47.7%) followed by Amhara 336 (45.8%). The religion of most respondents was Orthodox 490 (66.8)%, followed by Muslim161 (21.9%). The occupation of respondents was 442 (60.2%) house wife followed by government employee 135 (18.4%). The monthly income of the majority of the respondents were in the second (30.5%) was in the middle class (32.0%). Income level was classified by taking the minimum current Government employee salary as the first class (Table 1). Four hundred ninety nine (65.7%) of the respondents have more than five hundred birr per month.

**Table 1:** Socio-demographic characteristics of married women of Goba town, South East Ethiopia, September/ 2009

**Variable**	**Number**	**Percent**
**Age of the women**		
15-19	32	4.4
20-24	136	18.5
25-29	210	28.6
30-34	140	19.1
35-39	110	15.0
40-44	66	9.0
45-49	40	5.4
**Educational status**		
Illiterate	44	6.0
Able to read /write	32	4.4
Elementary	148	20.2
High school	343	46.7
12 and above	167	22.7
**Ethnicity**		
Oromo	350	47.7
Amhara	336	45.8
Gurage	29	4.0
Tigre	13	1.8
Others^*^	6	0.8
**Religion**		
Muslim	161	21.9
Orthodox	490	66.8
Protestant	74	10.1
Catholic	6	0.8
Others**	3	0.4
**Occupation**		
Government Employee	135	18.4
House wife	442	60.2
Merchant	104	14.2
Daily laborer	14	1.9
Student	36	4.9
Others***	3	0.4

*= Sidama, kembata, somali **=Kalhiwot, Missionary ***= Tella sailors, self employee002Ccpe.

### Utilization of LAPMs of contraceptives

The current utilization rate of LAPMs of contraceptives in the town was 64 (8.72%). Of these 48 (6.5%) were using Norplant, 11 (1.5%) IUD and 5 (0.7%) TL. One hundred thirty six (18.5%) of the respondents had ever used LAPMs. The methods ever used were 94 (12.8%) Norplant, 37 (5.0%) IUD and 5 (0.7%) TL. Two hundred nineteen 29.8% were pregnant and 195 (26.2%) had five or more pregnancies.

The utilization of LAPMs of contraceptives varies with change in the age group of respondents. The highest frequency of use was observed in the age group 25–29 years of age. However, there was a decrease in use of LAPMs as age of the women increase from 30–49 years.

Two hundred thirty nine 32.6% had no child and 155 (21.1%) had five and more than five children alive. Among 64 (8.72%) of current LAPMs users thirty-five 54.7% of users were using LAPMs of contraceptives for delaying pregnancy while twenty-six 40.6% of users were using LAPMs they did not want any more baby (Table 2).

**Table 2:** Percent distribution of current use, unmet need and demand for LAPMs by age, educational status and religion of married women of Goba town, South East Ethiopia, September/ 2009

**Variables**	**Using LAPMs %**	**Unmet need %**	**Demand %**
**Age**			
15-19	0.0	0.3	0.3
20-24	0.5	1.1	1.6
25-29	3.0	2.6	5.6
30-34	1.6	1.4	3.0
35-39	1.8	2.0	3.8
40-44	1.2	1.4	2.6
45-49	0.5	0.7	1.2
**Educational status**			
Illiterate	0.1	0.4	0.5
Able to read /write	0.4	0.5	0.9
Elementary	1.1	1.6	2.7
High school	4.5	3.8	8.3
12 and above	2.6	3.0	5.6
**Religion**			
Muslim	1.6	1.6	3.3
Orthodox	5.3	6.9	10.9
Protestant	1.5	0.8	2.3
Catholic	0.3	0.0	0.3
Others	0.0	0.0	0.0

### Demand and unmet need for LAPMs

The total demand for LAPMs of contraceptives in the town was found to be 18.12%. The unmet need for LAPMs of contraceptives was sixty-nine 9.4% (3.78% for spacing and 5.59% for limiting). Four 0.55% respondents who were pregnant at the time of the study had past unmet need as they have unintended current pregnancy of which two (0.27%) want spacing and two (0.27%) want limiting. Eighty-seven (11.8%) of the respondents want to limit their number of children. Of these 20 (2.72%) of them prefer to use LAPMs of contraception and were having unmet need for limiting. Four hundred ten 55.8% of the respondents were using short acting contraceptives including natural methods. Nevertheless, 33 (4.5%) of them did not want to use the method that they were using.

The total unmet need was higher in the age group 25–29 Years and it decline from age of 35 to 49 years. In addition, it was observed that there was declining of unmet need between ages 30–34 years. The unmet need for limiting increases with age, as age increase from 15 to 29 years. There was a decrease in pattern of unmet need for limiting and spacing as age increase from 30–49 years of age (Table 2).

### Associated factors utilization and unmet need for LAPMs

Two third (66.9%) of the respondents ever heard at least one LAPMs of contraceptives. The most ever heard LAPMs of contraceptive was Norplant 641 (87.3%). Four hundred forty five (60.8%) of the respondents have discussed with health professional about LAPMs. Six hundred thirty eight 86.9% reported, they knew where LAPMs of contraceptives were found in the town. Six hundred thirty six (86.6%) of the respondents have information about LAPMs from different sources. The major source of information was media 641 (87.3%) (Radio and Television) (Table 3). In the past few months, only two hundred fifty one 34.2% of the respondents discussed about LAPMs. Moreover, 179 (71.3%) have discussed with their husband and 90 (35.9%) discussed with their friends/neighbor. Three hundred fifteen (42.9%) of the respondents had never discussed about LAPMs of contraceptives with their husband /partner. Four hundred ninthly six (67.6%) of the respondents responded that their husband/partner approves partners using LAPMs. The decision of using LAPMs of contraceptive was mainly made by both partners together for 474 (64.6%) of the respondents (Table 3).

**Table 3:** Percentage of ever heard, intention to use, Knowledge of source, discussion with husband and source of information of LAPMs of married women of Goba town, South East Ethiopia September/ 2009

**Variables**	**Response**	**Frequency**	**Percent**
**Ever heard at least LAPMs**	IUD	491	66.9
	Norplant	641	87.3
	Tubal ligation	323	44.0
**Discussed with h/professional LAPMs**	Yes	289	39.4
	No	445	60.8
**Knows source of LAPMs**	Yes	638	86.9
	No	96	13.1
**Mentioned sources LAPMs**	Hospital	636	99.7
	Health center	17	2.6
	Clinic	12	1.9
	Private health facility	10	1.6
**Heard information of LAPMs**	Yes	636	86.6
	No	98	13.4
**Source of information of LAPMs**	Radio	389	53.0
	Television	252	34.3
	News paper	60	8.2
	Pamphlet	66	9.0
	Community events	488	66.5
	Health personnel	24	32.7
**Discussed with husband on LAPMs**	Not at all	315	42.9
	Once/twice	222	30.2
	More often	197	26.8
**Husband attitude to LAPMs**	Approves	496	67.6
	Opposes	38	5.2
	Don’t know	200	27.2
**Main decider on using LAPMs**	self	218	29.7
	Husband	42	5.7
	Both decide together	474	64.6

Among 677 (92.2%) who were not pregnant and not sure of their pregnancy status in the study period, 351(51.9%) wanted to become pregnant latter and 253 (37.4%) did not want any more. Of these seventy-five (21.4%) want to delay their next baby prefer to use LAPMs. The rest ninety (35.6%) want to use LAPMs to limit number of children. For majority of the respondents 532 (79.9%) their last pregnancy was intentional. Among current short acting contraceptive users 24 (68.6%) prefer to use Norplant. For half 12 (50.0%) of the non-users of Norplant the reason for not using Norplant was fear of complications. For those who want to use LAPMs, but are not using the methods they prefer the reasons for non-use are previous method inconvenient, health problem, husband/partner opposes and fear of complications (Table 4).

**Table 4:** Percentage of pregnancy status, plan for next baby, and proffered method, reason for none use of methods of married women of Goba town, South East Ethiopia, September 2009

**Variable**	**Response**	**Number**	**Percent**
	Yes	57	7.8
**Currently pregnant n=734**	No	669	91.1
	Not sure	8	1.1
**Pregnancy planned n=57**	Yes	50	87.7
	No	7	12.3
**Next baby is planned n = 677**	Latter	351	51.9
	Soon	73	10.8
	Not any more	253	37.4
**Preferred method to space n=351**	Short Acting	276	78.6
	LAPMs	75	21.4
**Preferred method limiters n = 253**	Short Acting	143	56.5
	LAPMs	90	35.6
	Nothing	20	7.9
**Want TL for limiters n= 253**	Yes	29	11.5
	No	224	88.5
**Reasons for not having TL n= 224**	Intercourse related	46	20.6
	Opposition of Tubal ligation	91	40.6
	Method related	23	10.3
	Health concern	43	19.2
	Prefer short acting methods	36	16.1

In the last few months only two hundred fifty one 34.2% of the respondents discussed about LAPMs. Discussion was made with husband/partner 179 (71.3%), father/ mother 4 (1.6%), daughter 7 (2.8%), health professionals 48 (19.1%) and friend/neighbor 90 (35.9%). Three hundred fifteen (42.9%) of the respondents had never discussed about LAPMs of contraceptives with their husband /partner. Four hundred ninety six (67.6%) of the respondents responded that their husband/partner approves partners using LAPMs. Thirty-eight 5.2% the decision of using LAPM of contraceptive was mainly made by both partners together for 474 (64.6%) of the respondents (Table 4).

Among those who were not pregnant and not sure 677 (92.2%) in the study period 351(51.9%) wanted to become pregnant latter and 253 (37.4%) did not want any more. Of seventy five (21.4%) who want to delay their next baby prefer to use LAPMs and ninety (35.6%) want to use LAPMs to limit number of children (Table 4).

Majority of the respondents 543 (80.2%) their last pregnancy was intentional. Among current short acting contraceptive users 24 (68.6%) preferred to use Norplant and for half 12 (50%) of the respondents reason for not using Norplant was fear of complications. IUD was not used in the past because of proven health problem 4 (57%) (Table 5).

**Table 5:** Percentage of last pregnancy intended, methods preferred & reasons for not using LAPMs by married women of Goba town, South East Ethiopia, September 2009

**Variable**	**Response**	**Number**	**Percent**
**Last pregnancy was planned n=677**	Yes	543	80.2
	No	134	19.8
**Method that have been used n=134**	Short term	92	68.7
	Natural methods	9	6.7
	IUD	5	3.7
	Norplant	25	18.7
	Tubal ligation	3	2.2
**Currently used Short Acting Method preferred n=410**	Yes	375	91.4
	No	35	8.6
**What LAPM was wanted n=35**	IUD	7	20.0
	Norplant	24	68.6
	Tubal ligation	4	11.4
**Reason for not using IUD n= 7**	Previously used methods inconvenient	2	28.6
	Proven health problem	4	57.1
	Partner/husband opposed	1	14.3
**Reason for not tubal ligation n= 4**	Fear of complications	3	75.0
	Partner/husband opposed	1	25.0
	proven health problem	6	25.0
**Reason for not using Norplant n=24**	Fear of complications	12	50.0
	Partner/Husband opposed	6	25.0

In order to see the association between unmet need for LAPMs and explanatory variables, binary logistic regression carried out to assess the statistical association of the dependent variable and the covariates. Those variable found to be statistically significant within the binary logistic regression model based on the COR and their p-value (p <0.05), were entered to the multivariable logistic regression model so that to come up with final predictors of utilization and unmet need of LAPMs contraception with their respective adjusted odds ratio. Ever use of LAPMs of contraceptives was found to be a strong predictor of current use of LAPMs, respondents who had ever used LAPMs of contraception were more than seventeen times more likely to use LAPMs than those who did not have ever used LAPMs [AOR= 17.43, 95% CI:9.19, 33.03]. The number of times discussion on LAPMs was made was also found to be predictor of using LAPMs of contraceptives, respondents who discussed more often were more than four times more likely to use LAPMs than those who have discussed once or twice about LAMPs of contraceptives [AOR= 4.6, 95% CI:1.72, 12.17]. Statistically significant association was not seen between age of women, number of children alive, educational status, religion, income of the family and other variables; and utilization of LAPMs of contraceptives (Table 6).

**Table 6:** Association of use of LAPMCs and socio-demographic characteristics and others related factors of married women of Goba town, South East Ethiopia, September 2009

**Variable**	**Using LAPMs**		**Crude**	**Adjusted**
	**Yes (%)**	**No (%)**	**OR**	**OR**
**Age**				
<30 years	26 (6.9)	352(93.1)	0.62(0.37,1.04)	0.48(0.16,1.47)
≥ 30 years	38(10.7)	318(89.3)	1.00	1.00
**Number of pregnancy**				
Zero	11(5.0)	208(95.0)	0.52(0.24, 1.13)	1.06(0.39, 2.90)
One/Two	16(8.9)	164(91.1)	0.96(0.47, 1.94)	0.86(0.31,2.38)
Three/Four	19(13.6)	121(86.4)	1.54(0.78, 3.06)	0.50(0.18, 1.38)
Five or more	18(9.2)	177(90.8)	1.00	1.00
**Number of children alive**				
Zero	13(5.4)	226(94.6)	0.58(0.27, 1.27)	1.50(0.14, 16.45)
One/Two	21(10.7)	176(89.3)	1.20(0.59, 2.45)	0.74(0.15,3.79)
Three/Four	16(11.2)	127(88.8)	1.27(0.60, 2.70)	0.68(0.17,2.75)
Five or more	14(9.0)	141(91.0)	1.00	1.00
**Educational status**				
Illiterate	4 (5.3)	72(94.7)	0.49(0.17,1.40)	0.93(0.26, 3.36)
Elementary	8 (5.4)	140(94.6)	0.50(0.23,1,09)	0.77(0.30, 2.01)
High school and above	52 (10.2)	458(89.8)	1.00	1.00
**Religion**				
Muslim	12(10.2)	149(92.5)	0.81(0.42,1.55)	***
Christian	52(9.1)	521(90.9)	1.00	
**Occupation**				
Government employed	17(12.6)	118(87.4)	1.69(0.94,3.05)	1.12(0.51, 2.47)
All others	47(7.8)	552(92.2	1.00	1.00
**Monthly income**				
< 500 Ethi. Birr	31(6.6)	439(93.4)	1.00	1.00
≥ 500 Ethi.Birr	33(12.5)	231(87.5)	**2.02(1.21,3.39)**	0.64(0.35, 1.16)
**Main decider**				
Self/Husband	11(4.2)	249(95.8)	1.00	1.00
Both together	53(11.2)	421(88.8)	2.85(1.46,5.56)	2.19(1.03, 4.65)
**Number of discussion**				
Once/Twice	5(1.6)	310(98.4)	1.00	1.00
More often	59(14.1)	360(85.9)	**10.16(4.03,25.64)**	**4.57(1.72, 12.17)**
**Husband opinion**				
Yes	59(11.0)	475(89.0)	4.84(1.92,12.25)	0.50(0.15, 1.73)
No	5 (2.5)	195(97.5)	1.00	1.00
**Ever use**				
Yes	49(36.6)	85(63.4)	**22.48(12.08,41.87)**	**17.43(9.19,33.03)**
No	15(2.5)	585(97.5)	1.00	1.00

*** - Variables with P-value > 0.05 were not entered in to regression model.

The intention to use LAPMs of contraceptives in the future was found to be a predictor of unmet need for LAPMs, respondents who were intending to use LAPMs were found about four times more likely to have had unmet need for LAPMs of contraceptives than those who did not intend to use [AOR=3.99, 95% CI:2.27, 7.02]. The other important predictor found was number of pregnancy, respondents who had five or more pregnancies were more than three times more likely to have unmet need than those who had not ever been pregnant [AOR= 3.67, 95% CI: 1.75, 7.71]. The other socio-demographic and behavioral factors were not found to have statistically significant association with unmet need for LAPMs of contraceptives (Table 7).

**Table 7:** Association of unmet need for LAPMCs and socio-demographic characteristics and others related factors in married women of Goba town, South East Ethiopia, September 2009

**Variable**	**Unmet need for LAPMs**		**Crude**	**Adjusted**
	**Yes (%)**	**No (%)**	**OR**	**Yes (%)**
<30 years	29(7.7)	349(92.3)	0.66 (0.40, 1.08)	0.99(0.41,2.39)
≥ 30 years	40(11.2)	316(88.8)	1.00	1.00
**Number of pregnancy**				
Zero	11(5.0)	208(95.0)	1.00	1.00
One/Two	17(9.4)	163(90.6)	1.97(0.90, 4.33)	2.41(0.96, 4.78)
Three/Four	12(8.6)	128(91.4)	1.77(0.76, 4.14)	1.71(0.72, 4.05)
Five or more	29(14.9)	166(85.1)	**3.30(1.60, 6.81)**	**3.67(1.75, 7.71)**
**Number of children**				
Zero	12(5.0)	277(95.0)	0.30(0.15,0.67)	0.25(0.02, 2.68)
One/Two	18(9.1)	179(90.9)	0.61(0.31,1.18)	0.50(0.12, 2.29)
Three/Four	17(11.9)	126(88.1)	0.82(0.41, 1.61)	1.01(0.34, 2.74)
Five or more	22(14.2)	133(85.8)	1.00	1.00
**Educational status**				
Illiterate	7 (9.2)	69(90.8)	0.93 (0.41, 2.14)	***
Elementary	12(8.1)	136(91.9)	0.81 (0.42, 1.57)	
High school and above	50(9.8)	460(90.2)	1.00	
**Religion**				
Muslim	12(7.5)	149(92.5)	0.73(0.381,1.395)	***
Christian	57(9.9)	516(90.1)	1.00	
**Occupation**				
Government				
employed			**1.80 (1.02, 3.16**)	1.64(0.91, 2.97)
	19(14.1)	116(85.9)		
All others	50(8.3)	549(91.7)	1.00	1.00
**Monthly income**				
< 500 Ethi. Birr	44(9.4)	426(90.6)	0.99 (0.59,1.65)	***
≥ 500 Ethi.Birr	25(9.5	239(90.5)	1.00	
**Number of discussion**				
Once/Twice	30(9.5)	285(90.5)	1.03 (0.62,1.69)	***
More often	39(9.3)	380(90.7)	1.00	
**Intension to use**				
Yes	50(16.3)	257(83.7)	**4.13(2.38,7.16)**	**3.99(2.27, 7.02)**
No	19(4.5)	403(95.5)	1.00	1.00

## Discussion

Information about LAPMs of contraceptives was high 86.6%. In Gondar town information of modern contraception was 74.9% [6] In EDHS 2011, the information about over all modern contraception was 97.4% the difference may be due to increased awareness of communities about contraception including LAPMs in the through time [2]. Having information on more than one LAPMs allows clients to choose the preferred method to use. The method mix of LAPMCs is required to increase the use rate of LAPMs [7]. The major source of information, media in this study could create an opportunity to transfer information [8]. The utilization rate of LAPMs of contraception in the town was 64 (8.7%). This was still small proportion of the utilization of overall modern contraception in the town 59.1%. This is in line with the DHS survey result in Kenya [9]. In other sub Saharan African countries the utilization rate of LAPMs fewer than five to seven percent despite their high effectiveness [3,10,11].

The utilization rate of each method in this study was IUD 11 (1.5%), Norplant 48 (6.5%) and TL 5 (0.5%). EDHS 2011 reported the utilization of LAPMs in urban areas was IUD 0.9%, Norplant 3.8% and female sterilization 1.5% [2]. Facility based study in Guatemala showed that the utilization rate of the three methods was 6%, 8% and 16% respectively [9]. This low utilization rate of IUD and Norplant in Goba town could be due to difference in study setting. A study on FP clients in Tehuledre showed that the utilization of Norplant and IUD was 39% and 8.7% respectively. The main reason for using LAPMs of contraceptive in Goba was higher for spacing 54% than limiting 40.6%. This difference could be due to the higher utilization of Voluntary Surgical Contraception (VSC) (52.3%) in Tehuledre [5]

Although IUDs were the most widely used LAPMs around the world, in this study it was found that the use rate of IUD was very small. IUD can provide effective protection for up to 10 years and could be used as alternative to sterilization for women who may regret after TL [12]. The reasons for non-use of IUD in the town were proven health problem; previously used methods inconvenient and husband /partner opposed. These were also the reasons mentioned in a study in Guatemala [9].

The currently used long acting contraceptive by respondents is higher for Norplant. This is in line with finding of EDHS [2]. Increasing the varieties of implant by incorporating Implanone and Jedelle may increase the opportunity to use LAPMs of contraceptives. They are very safe and convenient; save client time, effort, money, and ease clients load of health facilities as a single visit could provide long duration of protection [3,13]. The methods are cost effective and immediately reversible action in case on Norplant and IUD. This makes them the best-suited contraceptive for spacing as well as limiting [3,14-17].

In this study behavioral factors like the number of times discussions on the methods with husband/partner, ever use of any the LAPMs and main decider on using LAPMs were found to be predictors in using LAPMs of contraceptives. This could be due to the reason that those who have frequent discussion with husband/partner, ever used and making decision along with their partner have promotive behavior of using the methods. This could mean there is a need to work in this respect by responsible bodies including service providers.

The highest proportion of unmet need and LAPMs of contraceptive was in the age group 20–39 years. This may indicate that there is still a room for increasing the utilization of LAPMs of contraceptives in the town. In addition, more than half 60.2% of the respondents were housewife by occupation who may not have access to information and services of reproductive health.

The unmet need for LAPMs of contraceptives in this study was 69 (9.4%) which is very much less than the overall modern contraceptive unmet need in Oromia region. EDHS reported unmet need for all methods was 15%. The regional unmet need for contraception was 29.9%. In addition, in this study the unmet need for limiting is higher than the unmet need for spacing. EDHS reported unmet need for spacing was higher than for limiting [2]. USAID reported unmet need for limiting is higher than the unmet need for spacing in many African countries like Ghana, Malawi, Zambia, and Tanzania [18]. The reasons for unmet need for LAPMs were proven health concern, partner/husband opposition, fear of complication and previous methods were not convenient. In Ethiopia, these and other reasons are the factors for not using LAPMs of contraceptives [2,4]. This shows that there is still a need to provide training for providers on LAPMs, involving husbands/ partners in community education on LAPMs of contraceptives, preparing guidelines for providers and client screening for use like in case of IUD insertions. The significant association between grand multigravidas (Five or more pregnancy) and unmet need could mean that there was a need to provide appropriate service to the need of these groups.

LAPMs of contraceptives are also suitable methods for HIV infected individual to reduce mother to child transmission of HIV as they can avoid unintended pregnancy for couples who are living with HIV without restriction [3,8]. A study in Addis Ababa showed that people living with HIV also has high-unmet need for contraception [19]. LAPMs could provide safe and effective contraception. Implants are even more effective than IUD in protecting pregnancy. Furthermore, women who had not been pregnant before and breast feeding women from six weeks on wards can use them. Their other advantage is immediate return of fertility after removal [3]. Misconception about the use and effectiveness of LAPMs were over came by, transferring the correct knowledge of source and information about their use is of great importance [8]. In the past few months 34.2% of the respondents have had discussed about LAPMCs and for 71.3% of them the discussion was made with husband/partner. This may create better opportunity for decision. Use of LAPMs of contraceptives by couples through negotiation and influence of men favors joint decision making on use.

Timing and spacing pregnancies is necessary to improve the outcomes of pregnancy and childbirth for mothers and children. The reasons for not using LAPMs of contraception by those who want to use them include intercourse related, opposition to use, method related and health concern. EDHS and other studies found similar reasons for non-use of LAPMs [2,4,20]. This problem could be alleviated through evidence-based policies, guild lines, proper education of husbands, wives, service providers, and promotion of LAPMs of contraceptives through media and community events and community based activities like health extension workers.

## Conclusion

The utilization of LAPMs of contraceptives in the town was higher as compared to the results of other studies and EDHS 2005. Even though there is a good knowledge of LAPMCs in the town, since there are still client with unmet need for LAPMs for both spacing and limiting there is a need to promote the use of LAPMs of contraceptives. The unmet need for LAPMs in the town was indicative of the need to work on unmet need reduction. The reasons mentioned for non use and unmet need for LAPMs of contraception can be managed through proper training of service providers, better counseling skill on family planning, improving method mix, proper management of side effects as they appears and better supply of equipment and commodities.

## Competing interests

The authors’ declare that they have no competing interests.

## Authors’ contributions

AT has taken a lead role in writing the proposal, submission and follow up for ethical review, data collection, data entry, and writing of the preliminary results. GD, and MY participated in the planning of the study by giving constructive comments and idea. GD and MY have involved significantly in the analysis and writing of the manuscript through commenting. All authors read and approved the final manuscript.
